# An Audit Assessing Compliance With CURB-65 (Confusion, Urea, Respiratory Rate, Blood Pressure, and Age ≥65 Years) Documentation in Adult Patients Admitted With Community-Acquired Pneumonia at a Tertiary Institution in Trinidad and Tobago

**DOI:** 10.7759/cureus.112901

**Published:** 2026-07-18

**Authors:** Tarun V Ramlogan, Damion Basdeo, Rajiv Bhagaloo, Loren De Freitas

**Affiliations:** 1 Internal Medicine, Sangre Grande Hospital Campus, Sangre Grande, TTO; 2 Medical Sciences, The University of the West Indies, St Augustine, TTO

**Keywords:** clinical audit, community-acquired pneumonia (cap), continuing medical education (cme), curb-65 score, trinidad and tobago

## Abstract

Introduction

Community-acquired pneumonia (CAP) is a major cause of morbidity and mortality worldwide. Early severity assessment is essential in guiding decisions regarding hospital admission, level of care, and antimicrobial management. The CURB-65 (Confusion, Urea, Respiratory Rate, Blood Pressure, and Age ≥65 Years) score is a validated clinical prediction tool recommended by the National Institute for Health and Care Excellence (NICE) NG250 guidelines for assessing mortality risk in adult CAP patients. Despite its clinical relevance, documentation and utilisation of the CURB-65 score are often inconsistent in busy healthcare settings. This audit aimed to assess CURB-65 documentation in adult patients admitted with CAP at the Sangre Grande Hospital Campus (SGHC), Trinidad and Tobago, and the effectiveness of targeted educational interventions in improving adherence.

Methodology

A retrospective and prospective clinical audit was conducted in the Internal Medicine Department at SGHC. Adult patients aged ≥18 years admitted with CAP between March and May 2025 were included in the retrospective phase, while patients admitted between February and April 2026 formed the prospective post-intervention phase. Data were collected from paper and electronic medical records. Variables assessed included demographic characteristics, CURB-65 documentation, and score accuracy. The intervention included a CME sensitisation session on CURB-65 and NICE NG250 guidelines, followed by monthly reminders circulated through the departmental WhatsApp communication platform. Data were analysed using Microsoft Excel and SPSS 23. Chi-square testing was used to compare documentation rates pre- and post-intervention.

Results

In the retrospective phase, 67 patient records were analysed. CURB-65 documentation was identified in only 10.3% (n = 7) of cases, although all documented scores were calculated correctly. Following the intervention, 76 patient records were analysed during the prospective phase. CURB-65 documentation increased significantly to 43.4% (n = 33), with 90.9% (n = 30) of documented scores calculated correctly. Statistical analysis showed a significant improvement in documentation compliance after the intervention (*p* < 0.001). The majority of patients in both phases had CURB-65 scores of 0 or 1, suggesting that a substantial proportion may have been suitable for outpatient management depending on clinical judgement and comorbidities.

Discussion

This audit demonstrated poor baseline compliance with CURB-65 documentation at SGHC, consistent with findings from studies conducted in Oman, China, and Ireland. Education through CME sessions and regular reminders significantly improved compliance rates. The findings reinforce the importance of ongoing clinical education and guideline sensitisation in improving adherence to evidence-based practice. Despite the improvement, documentation rates remained below the NICE-recommended standard of 100%, indicating the need for further quality improvement measures, including electronic decision-support tools and institutional clinical pathways.

Conclusion

CURB-65 documentation for CAP patients at SGHC was initially suboptimal but improved significantly following targeted educational interventions and sensitisation. However, future interventions such as CURB-65 posters, incorporating the score into initial clerking documents and the Health Information System should be implemented as part of a formal improvement project.

## Introduction

Community-acquired pneumonia (CAP) is a significant cause of morbidity and mortality worldwide. Accurate severity assessment at the time of diagnosis is crucial to guide clinical decision-making regarding hospital admission and level of care. The National Institute for Health and Care Excellence (NICE) guideline NG250, published in 2025, updates and replaces previous NICE pneumonia guidance, including NG138. In this audit, NICE NG250 was used as the current reference standard for CAP assessment and CURB-65 (Confusion, Urea, Respiratory Rate, Blood Pressure, and Age ≥65 Years) documentation. This updated guideline also states that the score should be used in conjunction with clinical judgement. It is a validated severity and mortality prediction tool based on five parameters. The CURB-65 criteria are standardised as confusion, urea >7 mmol/L, respiratory rate ≥30 breaths per minute, low blood pressure defined as systolic blood pressure <90 mmHg or diastolic blood pressure ≤60 mmHg, and age ≥65 years [1-3]. Despite its importance, CURB-65 is often underutilised or incompletely documented in busy clinical settings. This audit aims to assess adherence to guideline-recommended documentation and use of the CURB-65 score in the management of adult patients with CAP at Sangre Grande Hospital Campus (SGHC).

The use of this clinically relevant score has been assessed in other parts of the world. For example, an audit in Oman found that the CURB-65 score was documented in only 2.3% (n = 4) of the study population. This audit was conducted at a tertiary institution that could benefit from an objective score, in addition to clinical judgement, to determine the need for outpatient, ward, or intensive care unit admission. The study concluded that adherence to the CAP severity index was very poor and recommended implementation of a local hospital-based integrated pathway to improve adherence to this clinically relevant, validated score [4].

Patient safety is the primary reason for improving CURB-65 documentation. The World Health Organization emphasises that "first, do no harm" is a fundamental principle of healthcare and that reducing avoidable harm requires reliable systems, processes, and clinical decision-making [5]. In patients with CAP, accurate severity assessment supports early recognition of higher-risk patients who may require inpatient care or escalation, while also supporting safe discharge planning for lower-risk patients when clinically appropriate. Therefore, the purpose of CURB-65 documentation is not simply bed management, but safer, more consistent, and evidence-informed patient care.

In addition to improving patient safety, consistent CURB-65 documentation may also be cost-effective by supporting appropriate use of hospital beds and alternative pathways such as same-day emergency care, virtual wards, or hospital-at-home services. However, these decisions must remain guided by clinical judgement, comorbidities, frailty, clinical risk, home safety, and the patient’s support network. A study performed in China showed that none of the 1230 inpatients with CAP had a documented CURB-65 score. After reviewing the notes, it was found that 1118 of the 1230 patients had a CURB-65 score of 0 or 1. As a result, these patients could possibly have been managed as outpatients, resulting in increased bed space and possible financial savings [6].

An Irish study determined the use of the CURB-65 score, which was found to be 7% (n = 2). Interestingly, another Irish study with an intervention showed a similar initial rate of 5.4%. After the audit, an intervention bundle was introduced over a period of approximately one year. A similar intervention could be utilised at the SGHC if this audit reveals poor compliance with CURB-65 documentation [7,8].

The importance of determining the level of care and need for admission for a patient with CAP is crucial for achieving the best clinical outcome and appropriate distribution of hospital resources. Hence, the CURB-65 score has significant benefits. Based on the literature, there are no studies assessing its use in Trinidad and Tobago or the wider Caribbean. This audit will provide some insight into the extent to which it is used at SGHC. Based on the findings, this can lead to data-based recommendations, implementation of hospital-based pathways, and use of the audit cycle to improve patient care.

## Materials and methods

Study setting

The study was conducted at the SGHC, Eastern Regional Health Authority, within the Medical Department. SGHC is a tertiary institution that serves the Eastern Regional Health Authority, one of the five Regional Health Authorities in Trinidad and Tobago.

Study design and study sample

This was a retrospective and prospective clinical audit evaluating the use and documentation of the CURB-65 score in adult patients admitted with CAP over a three-month period. The NICE guideline NG138 recommends the use of the CURB-65 score once it can be calculated. This was later updated in NICE guideline NG250, which is consistent and states that the score should be used in conjunction with clinical judgement. This is the standard utilised in this audit. Based on this guideline, the CURB-65 score should be documented for all patients with CAP.

The CURB-65 is a validated severity and predicted mortality assessment tool based on five parameters. The CURB-65 criteria have been standardised as confusion, urea >7 mmol/L, respiratory rate ≥30 breaths per minute, low blood pressure defined as systolic blood pressure <90 mmHg or diastolic blood pressure ≤60 mmHg, and age ≥65 years. This follows a point system, where one point is allocated to each parameter. A score of 0 or 1 is correlated with low risk, mortality of less than 3%, and outpatient management. A score of 2 is correlated with intermediate risk, mortality of 3-15%, and hospital admission. A score of 3 to 5 is correlated with high risk, mortality greater than 15%, and inpatient management with consideration of critical care intervention [1-3].

This retrospective and prospective clinical audit evaluated the documentation and accuracy of CURB-65 scoring among adults admitted with CAP. The sampling approach was modelled on the audit methodology described by O'Kelly et al., in which eligible patients were identified during defined pre- and post-intervention audit periods rather than through formal sample-size calculation [8]. In that study, the authors reviewed patients over two two-month periods, yielding 37 patients in the pre-intervention phase and 32 patients in the post-intervention phase. Similarly, in the present audit, all eligible adult patients admitted with CAP during the selected audit windows were included. However, to increase the number of cases available for analysis and improve the reliability of the findings, a three-month period was used for both the retrospective pre-intervention phase and the prospective post-intervention phase.

Target population

The target population comprised adult patients (≥18 years) admitted to the medical wards at SGHC with a diagnosis of CAP according to the NICE guideline NG250 (2025) [3].

Inclusion criteria

The inclusion criteria consisted of patients aged 18 years and older and patients admitted with a clinical or radiological diagnosis of CAP.

Exclusion criteria

The exclusion criteria consisted of patients with hospital-acquired pneumonia (onset >48 hours post-admission), patients under 18 years of age, patients transferred from other hospitals or wards, and those with aspiration pneumonia.

Data collection

Data were retrospectively and prospectively collected after the intervention from electronic and paper-based medical records, including clerking notes, observation charts, and discharge summaries. Data points included gender, length of stay, age, vital signs, urea level, mental status (confusion) using the Glasgow Coma Scale, CURB-65 score documentation, and whether the score was documented correctly.

Retrospective audit

Data were retrospectively collected from March to May 2025 (three-month period). This was done using the inclusion and exclusion criteria described above.

Intervention

The intervention consisted of two parts. Firstly, there was a Continuing Medical Education (CME) teaching session within the department, which occurred in January 2026. This involved a sensitisation session on the CURB-65 score, reference to the gold standard (NICE guideline NG250, 2025), and findings from the retrospective audit. This session included house officers, registrars, and consultants, all of whom are involved in the admission of patients. Secondly, monthly reminders of the CURB-65 score criteria were posted in the team's WhatsApp group. This group consisted of the same hierarchy of doctors who attended the CME in January 2026. The reminders were posted on the first day of February, March, and April 2026, with a reminder to utilise the score.

Prospective audit

After the CME teaching session, during which the staff were sensitised to the CURB-65 score, the gold standard stated in NICE guideline NG250, and the findings from the retrospective audit, the prospective audit commenced. This occurred immediately after the sensitisation session in January 2026. A reminder of the CURB-65 score was posted monthly on the team's communication platform (WhatsApp). The prospective audit took place over three months: February, March, and April 2026.

Data analysis

Data were compiled into an Excel spreadsheet (Microsoft Corporation, Redmond, Washington). The main analysis in this audit was the comparison between current practice and best practice, which should be 100% according to the updated NG250 [3]. Descriptive statistics were used to evaluate compliance rates, frequency of score documentation, and correlation with care decisions. Additionally, possible reasons for the lack of compliance were discussed. Quantitative data were analysed using Microsoft Excel and IBM SPSS Statistics for Windows, Version 23 (Released 2015; IBM Corp., Armonk, New York). The statistical test utilised was the chi-square test to compare observed categorical data.

Data management

All collected data were anonymised. No patient identifiers were included in reports or publications. Data were and will be stored securely and will be accessible only to the audit team.

## Results

Retrospective analysis

In total, 86 patients were found on the admission listing meeting the basic inclusion criteria from March to May 2025 (three-month period). Of the 86 patients, 67 files were obtained and analysed. The average age was 59.34 ± 17.18 years. The youngest patient was 20 years of age, and the oldest was 93 years of age. The majority were male (56.72%, n = 38), while 43.28% (n = 29) were female.

The aim of this paper was to investigate the percentage increase in documentation of the CURB-65 score after the intervention. However, it was found that initially only 10.30% (n = 7/67) documented the score, but interestingly, 100% (n = 7/7) of those documented scores were accurate (see Table 1). 

**Table 1:** Documentation of the CURB-65 score before intervention CURB-65: Confusion, Urea, Respiratory Rate, Blood Pressure, and Age ≥65 Years.

CURB-65 Score Documented	Frequency (n)	Percentage (%)
No	60	89.70%
Yes	7	10.30%
Total	67	100.00%
Correctly documented	7 out of 7	100.00%

Comparison of retrospective and prospective outcomes

Post-intervention documentation increased compared with that before the intervention (10.70% to 89.30%). This increase was also found to be statistically significant (p-value < 0.001) (see Table 2). These were all patients referred to the Medical Team for review regarding admission. However, the majority of patients had a CURB-65 score of 0-1 (before intervention: n = 52 out of 67, 77.61%; after intervention: n = 49 out of 76, 64.48%) (see Table 3). This indicated that, based on the score alone, these patients could possibly have been managed as outpatients with CAP. 

**Table 2:** Documentation of CURB-65 before and after the intervention (chi-square test: χ² = 27.21, df = 1, p = 0.000000182911). CURB-65: Confusion, Urea, Respiratory Rate, Blood Pressure, and Age ≥65 Years.

CURB-65 Documented	Before Intervention	After Intervention
Yes	10.70% (n = 7)	43.42% (n = 33)
No	89.30% (n = 60)	56.58% (n = 43)
Number of patients	67	76
Correctly documented	100% (n = 7 out of 7)	90.91% (n = 30 out of 33)

**Table 3:** Calculated CURB-65 scores for all the patients who met the inclusion criteria in both audit phases CURB-65: Confusion, Urea, Respiratory Rate, Blood Pressure, and Age ≥65 Years.

Pre-intervention Audit (N = 67)			Post-intervention Audit (N = 76)		
CURB-65	Frequency	Percentage	CURB-65	Frequency	Percentage
0	25	37.31	0	20	26.32%
1	27	40.30	1	29	38.16%
2	11	16.42	2	21	27.63%
3	3	4.48	3	6	7.89%
4	1	1.49	4	0	0.0%
5	0	0.00	5	0	0.0%

## Discussion

In the summary of the updated 2025 NICE guidelines for CAP, it is stated that the severity and mortality risk scores should be used in conjunction with clinical judgement for each patient. The severity score highlighted was the Pneumonia Severity Index (PSI), and the mortality score was CURB-65 (in the hospital) or CRB-65 (in the community). Particular emphasis is given to CURB-65 being used once it can be calculated. Considering that our setting is a tertiary institution, where a urea level can be obtained via the main laboratory or the Accident and Emergency point-of-care machines with quick turnaround times, this score can be calculated for all patients [2,3,9].

In our audit, we used the aforementioned NICE guideline NG250 (updated from NICE guideline NG138, 2019, for CAP) as the gold standard, and this is further supported by the British Thoracic Society guidelines [10]. However, NICE guideline NG250 (2025) is more recent than the British Thoracic Society guideline (2009). The NICE guideline would have taken into consideration more up-to-date literature when the recommendations were developed.

For this audit, our intervention consisted of a Continuing Medical Education (CME) sensitisation session and monthly reminders via the medical team communication platform (WhatsApp group). The sensitisation session was found to be effective in previous studies, such as the "intervention bundle" utilised in Ireland and published by O'Kelly et al. (2020) [8]. However, the sensitisation session was only one part of this intervention bundle. The authors also utilised technology through a smartphone application used on clinicians' iOS and Android devices. The smartphone application was incorporated primarily to promote antimicrobial stewardship in addition to documentation of the CURB-65 score. In this Irish study, documentation increased from 5.4% to 46.9%. This is similar to our study, where a simple sensitisation session and monthly reminders via WhatsApp increased documentation from 10.7% to 43.42% (p < 0.001). This reinforces the need for CME and ongoing teaching in the clinical setting to ensure that recommended and updated clinical practices are followed.

Our initial documentation findings were in keeping with the existing literature. Other developing nations, such as Oman, had low documentation rates (2.3%) for the CURB-65 score [4]. At a Chinese medical hospital, none of the 1230 inpatients with CAP had documented CURB-65 scores. Furthermore, 716 (58.2%) inpatients had a CURB-65 score of 0, and 402 (32.7%) had a score of 1, all of whom could potentially have received ambulatory treatment [6]. Additionally, another Irish study published in 2008 showed that 7% of patients with CAP had a documented CURB-65 score, highlighting the need for intervention even in the developed world. However, our study revealed a higher initial documentation rate for the CURB-65 score (10.7%). In the Irish study (2008), more than 50% of patients with a score of 0 or 1 were admitted and could potentially have received outpatient treatment [7]. A similar trend was observed in the Chinese study, where more than three-quarters of patients had CURB-65 scores of 0 or 1. This suggests that some patients may have been suitable for discharge with safety-netting advice or alternative pathways such as same-day emergency care, virtual wards, or hospital-at-home services. However, this should not be interpreted as evidence that these admissions were inappropriate, as CURB-65 does not capture all factors that influence admission decisions, including hypoxia, comorbidities, frailty, complications, social circumstances, home safety, and local service availability [6].

The gold standard, according to NG250, is for the CURB-65 score to be documented in 100% of CAP cases, including all five parameters, at the time of admission. Although the percentage of documentation increased, we observed mild inconsistency in correctly calculating the CURB-65 score. This was not the primary aim of our study; however, upon reviewing the documentation and recalculating the scores, it was found that 30 (90.91%) of the 33 (100%) documented scores were accurate. A study performed at a US university-affiliated community teaching hospital found an electronic version of CURB-65 to be more accurate. This was introduced because of missing data required to calculate the CURB-65 score and was found to be more accurate when the electronic version (eCURB) was utilised. This is a possible solution to the discrepancy in documentation observed when the scores were rechecked during this audit. It shows that although a statistically significant (p < 0.001) increase was observed in our study, caution must be exercised to ensure that the score is calculated correctly [11].

This emphasises the point that future interventions should move beyond education alone and include system-level changes. To ensure sustainability, the next phase should be developed as a formal quality improvement project in collaboration with the hospital's Clinical Governance and Quality teams. Proposed measures include adding CURB-65 to the admission clerking proforma, displaying posters in clinical areas, introducing HIS reminders where feasible, and providing recurring education during junior doctor induction [12,13]. A repeat audit should then assess whether these system-level interventions improve and sustain documentation rates. Moving forward, the audit team will liaise with the Clinical Governance and Quality teams to explore incorporating CURB-65 into the main medical admission clerking proforma. This would provide a structured prompt at the point of admission and may improve consistency of documentation. Its impact should be assessed in the next audit cycle as part of a formal quality improvement project.

However, it is important to note that there are other scores used to determine the severity of illness and the need for intensive care unit (ICU) interventions. A systematic review compared the quick Sequential Organ Failure Assessment (qSOFA) score with CURB-65 to determine the need for critical care intervention. This systematic review found that CURB-65 was more sensitive, whereas qSOFA was more specific [14]. These are some of the other tools and scores that may have been utilised in the Accident and Emergency Unit and by the medical team. Hence, this is one possible explanation for the lack of utilisation of the CURB-65 score.

Assumptions and limitations 

This was a retrospective and prospective review of clinical notes and did not hamper the care of patients in any way. Additionally, no invasive procedures were performed during this study that could have resulted in any physical harm to the patients. The term CAP was not used in this clinical setting; instead, lower respiratory tract infection (LRTI) was used. Hence, the cases were further reviewed to ensure that the definition of CAP was met. This was correlated with several guidelines, including the BTS 2009, NICE 2019 NG138, and NICE 2025 NG250 guidelines, to ensure that the diagnosis was consistent with CAP [1-3].

A further limitation was the assessment of the confusion component of CURB-65. In routine documentation, formal cognitive or delirium assessments were inconsistently recorded. The Glasgow Coma Scale (GCS) has limitations that should be acknowledged and is not a validated tool for diagnosing acute confusion or delirium; it should not be used as a substitute for the 4AT [15]. Therefore, where formal AMT or 4AT documentation was absent, the audit relied on documented clinical evidence of new disorientation or acute confusion. Future quality improvement work should incorporate the 4AT into admission documentation for patients in whom delirium or acute confusion is suspected.

In the CURB-65 score, confusion is defined according to the guideline criteria as an abbreviated mental test score of 8 or less, or new disorientation in person, place, or time. However, current delirium guidance supports the use of the 4AT as a rapid assessment tool for suspected delirium in most hospital settings outside critical care. Therefore, future admission documentation should consider incorporating 4AT assessment when acute confusion or delirium is suspected, rather than relying on non-specific measures of consciousness [16].

For the purposes of this audit, CURB-65 was considered to have been used only if the score was clearly documented in the clinical records. If the score was not documented, it was classified as not documented and presumed not to have been formally applied. Where CURB-65 was not recorded, the audit team retrospectively calculated the score using the earliest available clinical parameters from the patient's presentation. Blood pressure and respiratory rate were taken from the initial Accident and Emergency triage observations, while the urea value was taken from the first available blood test result during the admission. These values were used because they represented the earliest objective data available and were considered the most appropriate parameters for assessing CURB-65 at the point of presentation.

## Conclusions

This study identified poor baseline documentation of CURB-65 among adult patients admitted with CAP at the SGHC. Following a targeted intervention consisting of a CME sensitisation session and monthly WhatsApp reminders, CURB-65 documentation improved significantly. This demonstrates that simple educational measures can improve awareness and documentation practices in a busy acute medical setting.

However, documentation remained below the expected standard, indicating that education and reminders alone are unlikely to produce sustained improvement. Patient safety remains the primary reason for improving CURB-65 documentation, as structured severity assessment supports early recognition of higher-risk patients, appropriate escalation of care, and safe discharge planning for lower-risk patients when clinically appropriate. Any potential bed management or cost-effectiveness benefits should therefore be considered secondary to safe, evidence-informed clinical decision-making.

Future interventions should focus on sustainable system-level change. The audit team plans to liaise with the Clinical Governance and Quality teams to develop this into a formal quality improvement project. Proposed interventions include incorporating CURB-65 into the main medical admission clerking proforma, displaying reminder posters in the Accident and Emergency Department and medical admission areas, introducing hospital information system reminders where feasible, and providing recurring education during junior doctor induction. A further audit cycle should be completed after these interventions to assess whether documentation rates improve further and whether the improvement is sustained over time.
